# Supplemental Vitamin D3 for the Prevention of Bone Stress Injuries in Collegiate Athletes

**DOI:** 10.7759/cureus.83320

**Published:** 2025-05-01

**Authors:** Kevin Williams, Christian Askew, Daniel Hughes, Jeffrey A Guy, J. Benjamin Jackson III, Chase Gauthier

**Affiliations:** 1 Department of Orthopaedic Surgery, University of Alabama at Birmingham School of Medicine, Birmingham, USA; 2 Department of Orthopaedic Surgery, Prisma Health Midlands, Columbia, USA; 3 Department of Orthopaedic Surgery, University of Tennessee College of Medicine Chattanooga, Chattanooga, USA; 4 Department of Orthopaedics, Sanford Health, Fargo, USA

**Keywords:** athletes, ncaa athletes, stress fracture, vitamin d, vitamin supplementation

## Abstract

Background:Vitamin D is vital for bone mineralization and turnover. Athletes are prone to bone stress injuries (BSIs) when repetitive forces exceed the bone's remodeling capacity. Our study aims to determine if providing supplemental vitamin D to collegiate athletes reduces the incidence of BSIs in this population.

Hypothesis: Providing supplemental vitamin D to collegiate athletes will reduce the incidence of BSIs in this population.

Study design: This is a prospective cohort study, with a level of evidence of II, which means a lesser-quality prospective cohort with a retrospective comparative cohort.

Methods:Two hundred and forty-five collegiate athletes were enrolled from a single institution. Serum 25-hydroxycholecalciferol (25(OH)D) status was measured twice: once in August and once in February. All athletes were provided and subsequently supplemented with 50,000 IU vitamin D3 taken orally once per week for eight weeks. Athletes were monitored during the school year for BSIs. These data were then compared to retrospective stress fracture reports from 2010 to 2015, during which athletes' vitamin D was not screened nor supplemented.

Results: The athletes tested showed seasonal decreases in 25(OH)D levels, but these were consistent across variations in sex and sport. The overall incidence rate of fractures during the season receiving vitamin D supplementation (one of 245, 0.41%) was lower than the cumulative incidence rate of fractures during the five seasons with no supplementation (41 of 1974, 2.08%) with an absolute difference of 1.67%; however, the incidence rate difference was not statistically significant (p=0.073).

Conclusion:This study shows that vitamin D supplementation reduces the incidence of BSI in collegiate athletes, although not significantly so.Further studies are needed to characterize whether vitamin D supplementation can reduce fractures in collegiate athletes.

Clinical relevance: Vitamin D supplementation in collegiate athletes may reduce the overall incidence of BSIs in this cohort.

## Introduction

Vitamin D is a fat-soluble hormone that is produced endogenously when the skin is exposed to ultraviolet light. While it has many functions in the body, it is best known for its role in calcium homeostasis and bone mineralization. With sufficient calcium levels, the active form of vitamin D, 1,25-dihydroxycholecalciferol or 1,25(OH)2D, promotes bone remodeling and mineralization through osteoblast activation and osteoclast maturation [1]. Clinically, vitamin D status is determined by serum measurements of the inactive form of vitamin D, 25-hydroxycholecalciferol or 25(OH)D. Sufficient vitamin D status is important for maintaining skeletal integrity. Previous research has shown hypovitaminosis D is prevalent among collegiate and professional athletes with ranges between 33% and 90% [2-6]. 

As bone experiences mechanical loading, there is a resultant microfracture that forms in the bone matrix. Subsequent activation of the remodeling process helps to repair the microdamage and ensures adequate bone adaptation to skeletal loading [7]. When repetitive mechanical stress exceeds the bone's repair capacity, the excess microdamage results in the formation of a bone stress injury (BSI) [8]. BSIs are overuse injuries commonly seen in athletes that develop from increased training and/or reduced recovery time. Accounting for 10-20% of all sports-related injuries, BSIs are painful and can hinder athletic performance [9,10].

Current research in orthopedics and sports medicine seeks to evaluate supplemental vitamin D's protective properties on BSIs, with some suggestions that inadequate vitamin D levels may predispose an athlete to develop BSIs [11-14]. Previous studies found strong correlations between stress fracture incidence and vitamin D status among military recruits, with a higher rate of stress fractures occurring in military recruits who demonstrated low vitamin D levels [15,16]. Lappe et al. determined vitamin D and calcium supplementation was protective against BSI development in female Navy recruits [17]. However, there is little data in the literature examining vitamin D supplementation as a preventive measure against BSI formation in athletic cohorts. The current study aims to determine if providing the National Collegiate Athletic Association (NCAA) Division I collegiate athletes with supplemental vitamin D can reduce the incidence of stress fractures in this population. The secondary objective of this study is to determine if providing NCAA Division I collegiate athletes with supplemental vitamin D can reduce the prevalence of abnormal vitamin D levels compared to athletes who were not provided supplemental vitamin D. We hypothesize that athletes who are provided supplemental vitamin D will have a lower rate of stress fractures and abnormal vitamin D levels compared to athletes who were not provided supplemental vitamin D.

## Materials and methods

This study was approved by the Institutional Review Board of Prisma Health Midlands (approval number: Pro00057204) on April 10, 2016. Informed written consent was obtained from all subjects who voluntarily enrolled in this study. The retrospective portion of the data collection did not require consent as all data points were de-identified and no individuals were contacted.

Subject enrollment

NCAA Division I varsity collegiate athletes from the University of South Carolina in Columbia, South Carolina, were invited to participate in the current study during their required pre-participation examinations (PPEs) before the 2016-2017 athletic season. Athletes were screened using inclusion and exclusion criteria, and those who were under the age of 18 at enrollment, were pregnant, or had a current diagnosed BSI based on evaluation by a team athletic trainer were excluded from the study. A total of 245 athletes from 17 different varsity sports provided informed written consent and were subsequently enrolled in the study. 

Upon consent, all enrolled subjects were asked to complete a demographic questionnaire regarding age, height, weight, sex, and sporting team. Additionally, subjects responded to questions concerning their current vitamin D supplementation habits, any history of BSI, and any history of low bone density. Subject-reported responses indicated 81 male and 164 female athletes with a cohort mean age at enrollment of 19.6 years of age (±1.32). 

Vitamin D status was categorized based on guidelines from the Endocrine Society: sufficient status with serum 25(OH)D 30 ng/mL and abnormal status with serum 25(OH)D <30 ng/mL [18]. Subjects had serum 25(OH)D levels assessed in August during required PPEs and in February following their return from winter break. Two hundred and forty-five subjects completed the testing in August 2016. However, only 191 subjects completed both testing periods (August and February). 

Following testing, all 245 subjects were provided supplemental vitamin D3 in the form of 50,000 IU capsules to be taken orally once per week for eight weeks (Bio-Tech Pharmacal, Inc., Fayetteville, AR, USA), a commonly prescribed dose and treatment regimen which has been utilized in previous studies [14,19]. Subjects whose serum 25(OH)D status was greater than 70 ng/mL at either testing period were excluded from supplementation due to concerns of potential vitamin D toxicity. Subjects were monitored during all sports-related activities by the university's athletic training staff for any signs of BSI development. Subjects with complaints of BSI symptoms would receive appropriate medical care from the team's training staff and sports medicine physicians. 

Retrospective data collection

The control group for this study was comprised of retrospectively collected data from a historical cohort at our institution. No subjects were contacted or enrolled for the retrospective data collection phase. The incidence of BSI among collegiate athletes from the same institution from 2010 to 2015 was obtained from records kept by the university's athletic department. All data was de-identified and sorted by sports team and year. Additionally, vitamin D serum levels were compared to a historic control of vitamin D serum from athletes in five high-risk sports during the 2015-2016 athletic season. In this cohort, vitamin D levels were collected in both August 2015 and February 2016.

Statistical analyses

Key variables in the analysis included the incidence of BSIs and abnormal vitamin D levels for both the prospective experimental group and retrospective control group. Continuous variables are reported as a mean and standard deviation, while categorical variables are reported as a value and proportion. Comparisons of continuous variables were calculated using Student's t-test, while comparisons of categorical variables were calculated using Fisher's exact test. Graphs and calculations were performed using the Microsoft Excel Data Analysis toolkit (Microsoft Corp., Redmond, WA, USA). All p<0.05 were considered significant.

## Results

25(OH)D serum variation by academic year

**Figure 1:**
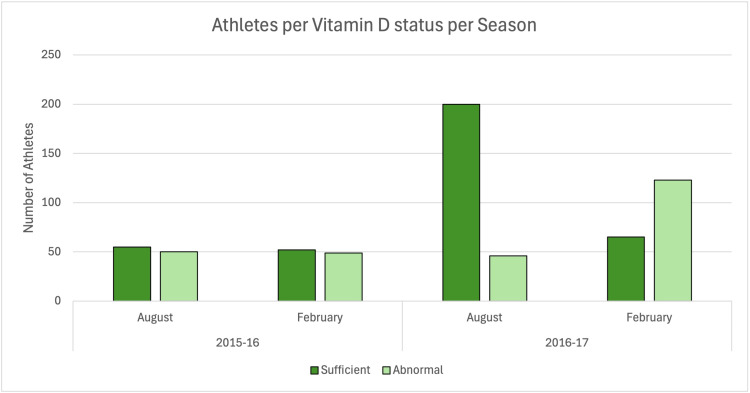
Total sum of athletes in each test year with sufficient and abnormal serum 25(OH)D levels. 25(OH)D: 25-hydroxycholecalciferol

Vitamin D serum levels from the 2016 to 2017 season were compared to vitamin D levels from the 2015 to 2016 season. As shown in Figure 1, in the 2015-2016 season, in which athletes were not given supplemental vitamin D, 54.46% (61 of 112 athletes) had sufficient vitamin D in August, and 53.85% (56 of 104) had sufficient vitamin D in February. In comparison to our test year of the 2016-2017 season, in which all participating athletes were given supplemental vitamin D, 82.04% (201 of 245 athletes) had sufficient vitamin D in August, and only 35.08% (67 of 191 athletes) had sufficient vitamin D in February. 

25(OH)D serum variation by sport, location, and sex

**Figure 2:**
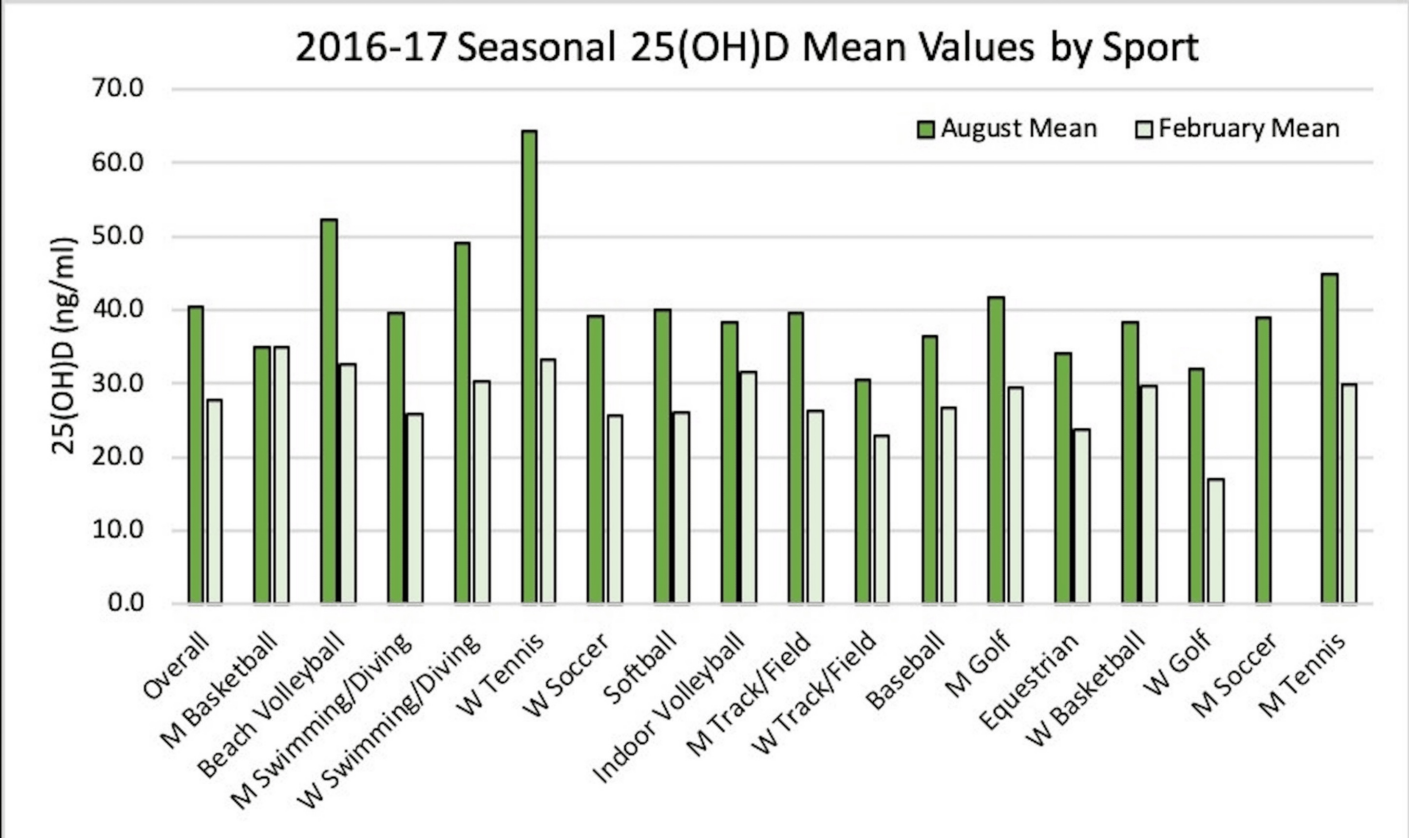
Average 25(OH)D values divided by sport and compiled overall for the 2016-2017 season where athletes were supplemented with vitamin D. 25(OH)D: 25-hydroxycholecalciferol

Across all sports in the 2016-2017 season, serum 25(OH)D levels in August 2016 were an average of 40.4 ng/mL with a standard deviation of 14.3. In February 2017, serum levels were an average of 27.7 ng/mL with a standard deviation of 8.85. In August 2016, no sport had an average serum 25(OH)D level in the abnormal range. In February, 11 of 16 sports, including men's swimming and diving, women's soccer, softball, men's and women's track and field, baseball, men's golf, equestrian, women's basketball, women's golf, and men's tennis had average vitamin D levels in the abnormal range. As seen in Figure 2 of a comparison of August levels of 25(OH)D to February levels, every sport except for men's basketball and women's tennis had a statistically different average serum 25(OH)D level. 

Male versus female sports were analyzed and showed no statistically significant difference in average serum vitamin D levels in both August 2016 and February 2017. Male athletes had an average of 38.9 ng/mL 25(OH)D in August (standard deviation of 9.7) and 28.1 ng/mL 25(OH)D in February (standard deviation of 7.5). Female athletes had an average of 41.1 ng/mL 25(OH)D in August (standard deviation of 15.4) and 27.5 ng/mL in February (standard deviation of 9.4). Additionally, both male and female athletes had a statistically significant decrease in 25(OH)D within their respective groups measured in February as compared to the prior level obtained in August. 

Given the known effects of sun exposure on 25(OH)D levels, athletes were separated into indoor and outdoor sports. Outdoor sports had an average of 40.3 ng/mL 25(OH)D in August (standard deviation of 15.74) and 26.8 ng/mL in February (standard deviation of 8.65). Indoor sports had an average of 40.5 ng/mL 25(OH)D in August (standard deviation of 10.17) and 28.3 ng/mL in February (standard deviation of 8.17). The difference between these two groups was not significantly different during August and February. 

Stress fracture outcomes

For the incidence of the BSI section of the study, 245 patients were included in our experimental group and 1974 in our control group. Table 1 includes the list of sports teams recruited and the number of athletes per team as well as stress fracture events per sport.

**Table 1:** Total and sport specific SF incidence for the 2010-2015 seasons and the 2016-2017 season. SF: stress fracture

	2010-2015 (5 seasons)			2016-2017 (1 season)		
Team	SF events	Athletes	SF percent	SF events	Athletes	SF percent
Women's track and field	12	350	3.43%	0	19	0.00%
Women's soccer	9	148	6.08%	0	22	0.00%
Men's track and field	5	189	2.65%	1	22	4.55%
Men's basketball	3	68	4.41%	0	10	0.00%
Men's soccer	3	131	2.29%	0	7	0.00%
Indoor volleyball	2	80	2.50%	0	18	0.00%
Women's basketball	2	64	3.13%	0	10	0.00%
Beach volleyball	1	43	2.33%	0	21	0.00%
Men's swimming and diving	1	116	0.86%	0	8	0.00%
Women's tennis	1	46	2.17%	0	7	0.00%
Baseball	1	172	0.58%	0	25	0.00%
Men's tennis	1	46	2.17%	0	6	0.00%
Women's swimming and diving	0	190	0.00%	0	23	0.00%
Softball	0	109	0.00%	0	19	0.00%
Men's golf	0	0	0.00%	0	3	0.00%
Equestrian	0	186	0.00%	0	18	0.00%
Women's golf	0	36	0.00%	0	7	0.00%
Total incidence	41	1974	2.08%	1	245	0.41%

Vitamin D testing and supplementation

As shown in Table 2, during the 2016-2017 season, in which athletes were supplemented with vitamin D and monitored, there was only one BSI reported out of 245 athletes (0.41%). This stress fracture was in men's track and field. In the five seasons from 2010 to 2015, there were a total of 41 BSIs out of 1974 athlete years (2.07%). The overall incidence rate of fractures during the season receiving vitamin D supplementation was lower than the cumulative incidence rate of fractures during the five seasons with no supplementation with an absolute difference of 1.67%; however, the incidence rate difference was not statistically significant (p=0.079). A post hoc power analysis demonstrated this study has an 83.9% power to detect a 20% difference in the rate of BSIs between our two study groups.

**Table 2:** 2×2 contingency table showing the incidence of SF in vitamin D-supplemented and vitamin D-non-supplemented athletes. SF: stress fracture

	SF positive	SF negative	Total
2010-2015 (no vitamin D supplement)	41	1933	1974
2016-2017 (vitamin D supplemented)	1	244	245

The highest number of cases per sport was in women's track and field with 12 reported BSIs from 2010 to 2015. The second and third highest BSI rates were in women's soccer with nine cases and in men's track and field with five cases. After adjusting for a percentage of total athletes tested, since the track and field team is significantly larger than most other teams, the top percentage incidence of BSIs was in women's soccer at a rate of 6.08% from 2010 to 2015. The second and third highest percent incidences were in men's basketball at 4.04% and women's track and field at 3.43%. Of note, men's golf was included in the study season of 2016-2017 but was not included in prior data sets. Additionally, beach volleyball only has data for the 2014 and 2015 seasons. 

Side effects of vitamin D supplementation and survey results

During the study, athletes did not report any side effects from the vitamin D supplementation. Additionally, prior to the start of the study, the athletes filled out a survey for demographic data as described above. This revealed that some athletes were already taking vitamin D before this study. Thirty-three of 245 athletes (13.5%) were taking vitamin D supplementation in some form, either in addition to or part of a multivitamin. There was no significant difference in the rates of vitamin D deficiency at the start of the study in August between athletes taking vitamin D as a multivitamin (5/33=15.1%) and those not taking a vitamin D supplement (39/212=18.4%) (p=0.68). There was also no significant difference in the rates of vitamin D deficiency at the end of the study in February 2017 between athletes taking vitamin D prior to the start of the study (17/33=51.5%) and those who only took vitamin D during the study period (107/162=66%) (p=0.34). 

## Discussion

Serum 25(OH)D levels

This study aimed to see if vitamin D supplementation reduced the incidence of BSI as its primary aim. This study was a follow-up study from a similar study we performed in 2015-2016 [20], and an abstract from the current study was previously published prior to the manuscript [21]. We noted positive results when selectively supplementing high-risk sports for BSI [21]. Given those preliminary results, we expanded this follow-up vitamin D study to all of our Division I collegiate athletes. Overall, there was a decrease in the overall rate of BSI in those who received supplemental vitamin D compared to those who did not, mirroring the results of a previous randomized controlled trial conducted by Millward et al., although this difference was not statistically significant [14]. Additionally, of patients who were given supplemental vitamin D, approximately 64.9% had abnormal vitamin D levels following their supplementation regimen. This is in contrast to patients who did not receive vitamin D supplementation, of which only 46.1% of athletes had abnormal vitamin D levels. 

It is well studied that there is seasonal variation in 25(OH)D levels due to (1) less ultraviolet light exposure in the northern hemisphere and (2) less time spent outdoors in cold weather. It is unknown, however, if this biannual variation would lead to increased BSI in collegiate athletes. We found that 16 weeks of vitamin D supplementation throughout the academic year was insufficient to prevent abnormal serum vitamin D levels in our cohort. Also, athletes who were already supplementing their diet with vitamin D did not have any difference in levels of vitamin D deficiency. It's important to note that the lower February vitamin D levels are less clinically relevant as compared to the outcome of decreased BSI. Confounding factors between these two years include fewer sports tested during the 2015-2016 year (five total) compared to 17 sports tested in the 2016-2017 year. An explanation for this observed finding may be that in August, athletes had likely had summer training and more exposure to the sun over the summer. The February serum levels reflect time spent during the sport, as well as the lower sun exposure in fall and winter and the time needed indoors to study for classes, with these factors collectively leading to lower sunlight exposure time. 

There was no difference between the outdoor and indoor athlete groups in serum vitamin D concentration in both August and February of the study period. This may indicate that exposure to the sun while training and performing their sport is less significant than other factors affecting 25(OH)D serum levels. It should be noted, however, that nearly all sports, including football, have the ability to perform a significant amount of training indoors due to facilities that provide cover from the sun and other aspects of the weather. This factor was not accounted for in our study and could serve as a significant confounding variable.

Additionally, there was no significant difference in serum vitamin D concentrations between male and female athletes for both August and February, and both decreased at a similar rate in the winter months. This finding may indicate that male and female physiology does not impact the 25(OH)D levels measured as much as other factors, such as sunlight exposure. Another variable could have been the athlete's diet and whether or not they were getting enough calcium, phosphorus, and vitamin D, all of which may ultimately affect vitamin D utilization and metabolism [21].

BSI outcomes

The outcome of BSIs was lower for the 2016-2017 season as compared to the 2010-2015 seasons. However, the difference was not statistically significant likely due to low incidence. This suggests that vitamin D supplementation may reduce the overall incidence of BSI for collegiate athletes, although further data is needed to validate this claim. As expected, from 2010 to 2015, sports with higher physical contact to playing surfaces had the highest absolute value and percentages of BSI. Athletes in these particular sports may also be subject to certain styles of team practices, training over the seasons, and personal diet, all of which may additionally contribute to the observed findings. These results can be further solidified by having additional years of test data or having additional institutions repeat the study with similar findings. 

Although athletes who took vitamin D supplementation had a lower level of BSIs compared to those who did not take vitamin D supplementation, levels of 25(OH)D do not appear to be directly correlated with BSI, as the majority of athletes who consumed vitamin D supplementation had abnormal 25(OH)D levels in February, but only one BSI over the entire cohort. However, given the relatively benign side effects of vitamin D supplementation and that it is generally well tolerated, we recommend that collegiate athletes supplement with vitamin D, especially if they are in higher-impact sports with a significant amount of mechanical load involved [17]. Further experimentation with a larger sample size, and therefore a higher-powered study, may be warranted.

There are several limitations to this study. The data utilized in this study was collected in both a retrospective and prospective fashion, which may introduce the potential for bias. Also, data for the study was collected from 2010 to 2017, which may limit its applicability to current collegiate athletes, thereby limiting the relevance of the results to modern audiences. Additionally, rates of adherence to the prescribed vitamin D supplementation protocol were not collected, with incomplete adherence to the prescribed protocol potentially directly affecting the results of this study. Despite these limitations, this study demonstrated that vitamin D supplementation reduced the incidence of BSIs in college-level athletes, although this result did not reach statistical significance. 

## Conclusions

Our data demonstrated that athletes who received supplemental vitamin D had a lower incidence of BSIs compared to athletes who did not receive supplemental vitamin D, but the reduction did not reach statistical significance (p=0.07). However, given the low risk, low cost, and ease of this intervention, we would recommend considering vitamin D supplementation in elite athletes and future, large studies to further evaluate its efficacy.
